# A rare lipoma site in a 1-year-old boy

**DOI:** 10.1093/jscr/rjab447

**Published:** 2021-10-28

**Authors:** Gulan Maree, Bardisan Gawrieh, Ammar Omran, Wajih Ali

**Affiliations:** Pediatric Surgery Department, Tishreen University Hospital, Lattakia, Syria; Pediatric Surgery Department, Tishreen University Hospital, Lattakia, Syria; Pediatric Surgery Department, Tishreen University Hospital, Lattakia, Syria; Pediatric Surgery Department, Tishreen University Hospital, Lattakia, Syria

## Abstract

Lipomas are benign tumors composed of mature adipocytes. A 1-year-old-male baby was admitted to the hospital with a history of an abdominal mass, which had been incidentally detected by a local pediatrician. upon examining the child for a complaint of distention and chronic diarrhea. An ultrasound and computed tomography scan were performed, and findings revealed a fat mass, which was confirmed by laparotomy and microscopic results. At laparotomy, a soft yellow mass was found attached to the mesentery of the jejunum. The mass was enucleated without anastomosis, and the patient was followed up 4 months postoperatively with regular clinical examination and abdominal ultrasonography. This case was examined and reported in this study because lipomas are very rarely present in the mesentery of the intestine, especially at this early age.

## INTRODUCTION

Lipomas may be found anywhere in a child’s body but rarely so in the mesentery of the intestine. To date, fewer than 50 cases of mesenteric lipoma have been reported in the literature published in English, with nearly a half occurring in children [1–3].

Lipomas are usually asymptomatic until they grow large enough to cause symptoms, such as pain or constipation due to partial or complete obstruction or volvulus. Although, with relation to the all said cases, no malignant tumors are reported, the preferred treatment for lipoma is complete resection with or without bowel resection [3]. Laparotomy and histology are the definitive diagnoses [2].

## CASE REPORT

A 1-year-old-male baby was presented to the hospital with a history of an abdominal mass. A local pediatrician in the outpatient clinic incidentally discovered it when he was examining the child for a complaint of distention and chronic diarrhea.

The child was referred to the hospital and was admitted to the Pediatric Surgery Department. Physical examination indicated the mass was painless and mobile. The palpable mass was nontender and extended from the upper left quadrant to the umbilicus. Vital signs were normal, and the child was asymptomatic with a good appetite. He did not complain of vomiting, constipation or abdominal pain. His weight at admission was 9 kg. Laboratory tests were normal. Abdominal ultrasound showed a hyperechoic soft tissue lesion with a maximum diameter of ~10 cm, pushing the intestine to the right without obstruction of the intestinal lumen.

**Figure 1:**
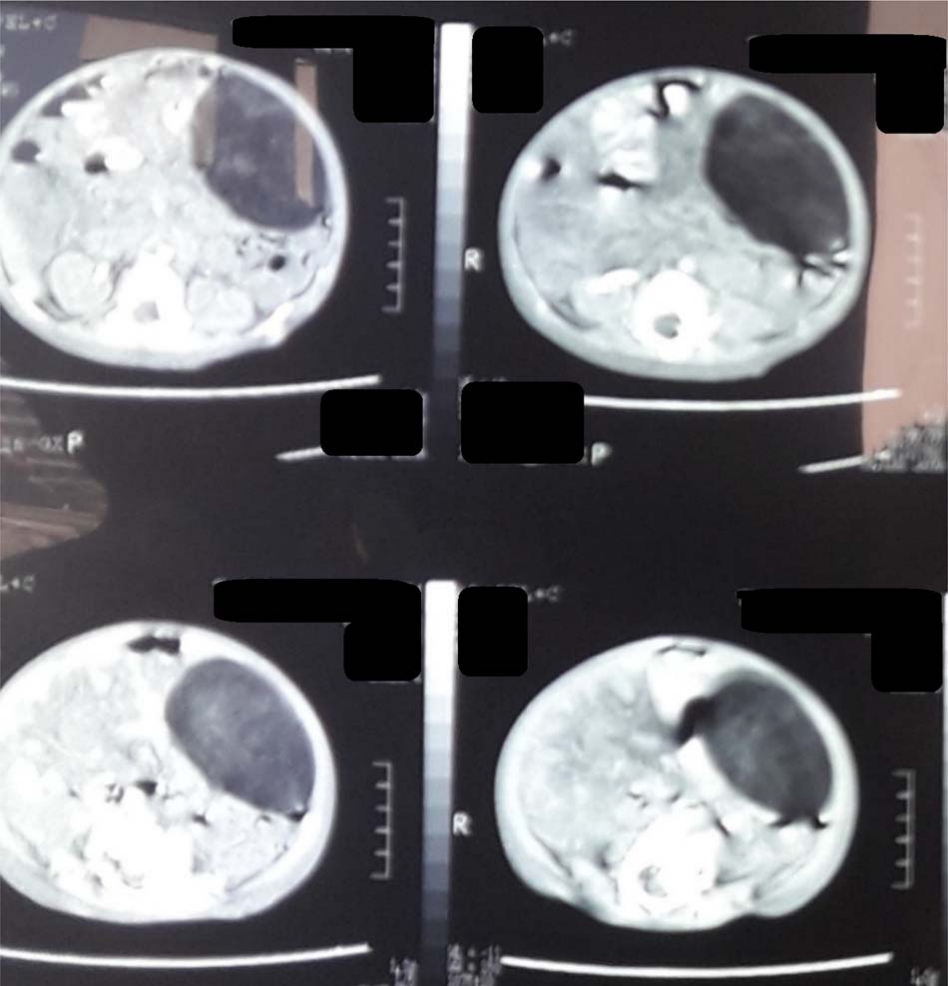
CT scan of the abdomen revealed a fat density mass with defined margins filling the left abdomen and shifting the small bowel to the right.

**Figure 2:**
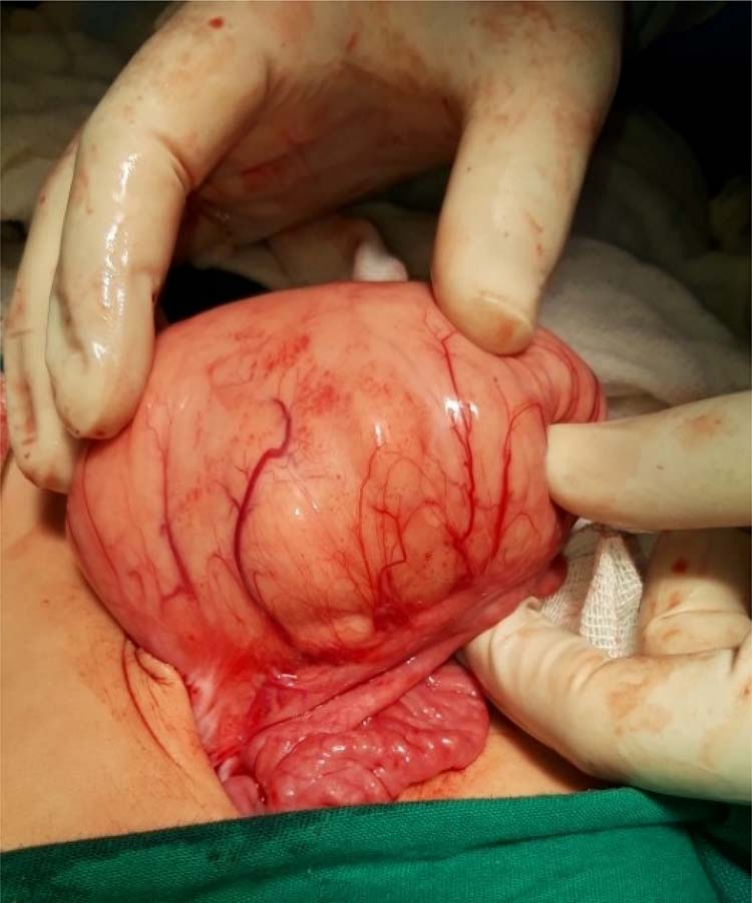
Mass adherent to the bowel and mesentery at exploration.

**Figure 3:**
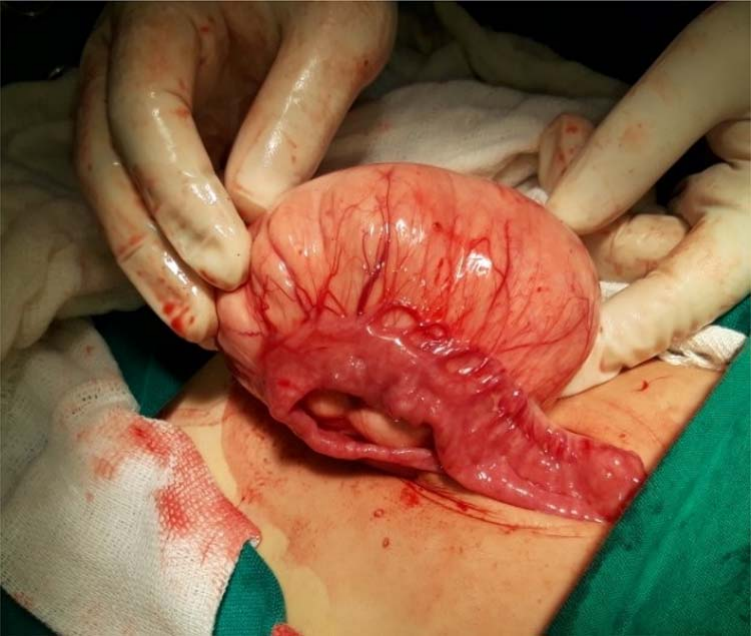
Appearance of the mass at exploration.

**Figure 4:**
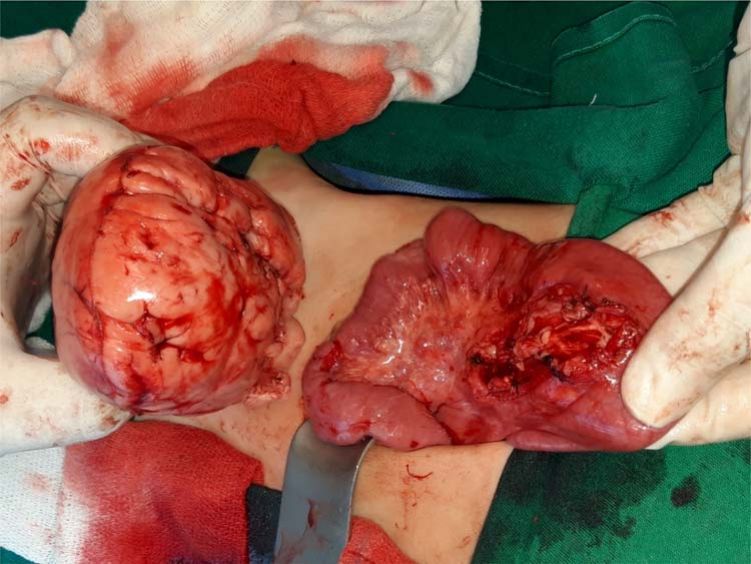
General appearance of the mass after excision.

**Figure 5:**
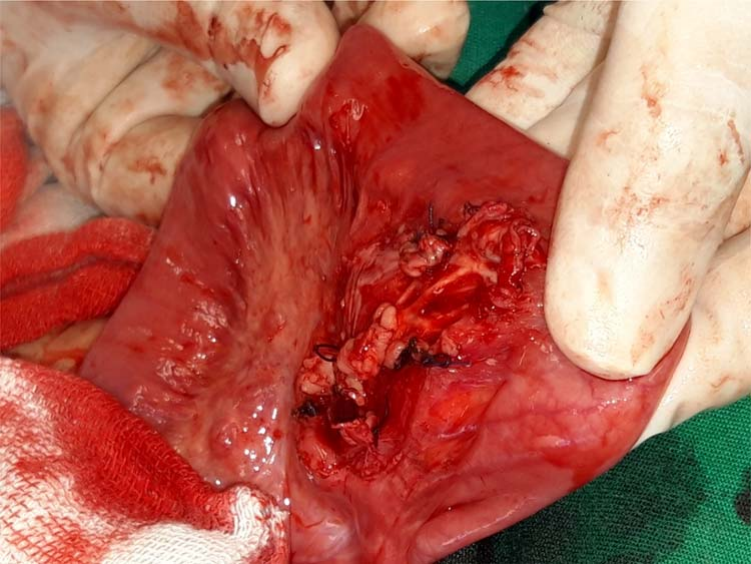
Appearance of the mesentery after the mass’s excision.

**Figure 6:**
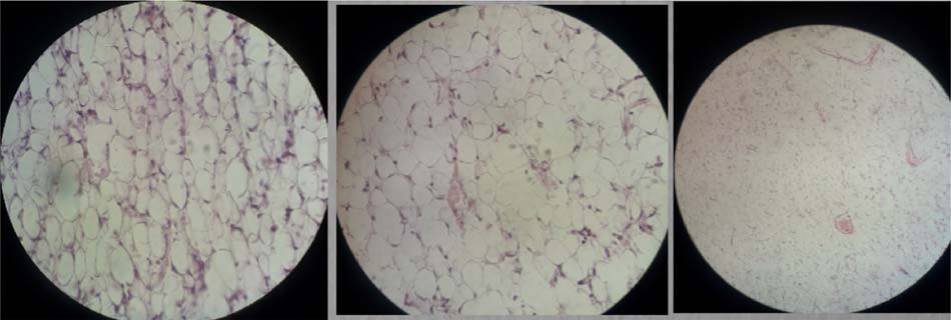
Histopathological examination showed mature fat cells and no cellular atypia.

Computed tomography (CT) of the abdomen revealed a homogeneous, well vascularized mass measuring 9.4 × 10.6 cm and encapsulated with thin septation in its medial part. The mass was located in the upper left quadrant and extended to the pelvis with an oval appearance (Fig. 1). Other abdominal findings were normal. Under general anesthesia, an incision was made in the midline above the umbilical region. The findings were a soft, yellow mass with defined margins, originating from the small intestine’s mesentery, 30 cm from Treitz ligament (Figs 2 and 3). Complete excision of the mass was performed without intestinal resection (Figs 4 and 5). Histopathology showed a normal adipose tissue composed of mature adipocytes with no evidence of mitosis or nuclear atypia, which confirmed the presence of the mesenteric lipoma (Fig. 6). Oral feeding was started 2 days after the operation, and the patient stayed in hospital for 5 days. He was discharged in a good condition with normal bowel movement. The child was evaluated 4 months after the procedure. He had no complains, and abdominal ultrasound revealed normal findings.

## DISCUSSION

Lipoma is a fatty tissue composed of mature cells and it has a benign behavior. It is more common in adults aged 40–60 than in children, and it rarely occurs in children under 10 years of age [3]. The lipoma in our patient was found in the mesentery of the jejunum about 30 cm from the Treitz ligament. In the medical literature, almost 18 cases of mesenteric lipomas in children have been reported, out of which just 2 were in the jejunal mesentery. The first involved the excision of an 11 × 9 × 5-cm mass from a 2.5-year-old male and the other concerned the excision of a 16 × 15 × 7.5-cm mass along with a segment of adjacent bowel from a 14-year-old girl [1, 2, 4, 5].

Until now, the causes of lipoma are not clear but some factors have been reported to be predisposing to the growth, such as diabetes mellitus, hypercholesterolemia, obesity, familial tendency, radiotherapy, chromosomal translocation and trauma [3, 6]. In our case, the medical history included lactose intolerance without the presence of diseases predisposing to lipoma as in adults.

Lipomas are slow growing and they do not penetrate the surrounding organs, but some may cause symptoms when the tumor grows too large and compresses the adjacent organs [6, 7]. The mass in our patient was 9 × 11 cm, movable, soft, asymptomatic and incidentally detected during examination of another complaint.

Ultrasound imaging can be used to distinguish lipomas from mesenteric cysts. Not to mention that it is better to perform an abdominal CT scan to distinguish lipoma from other echogenic tumors [3, 8]. The other masses that must be considered in differential diagnoses include dermoid cyst, omental and mesenteric cysts, intestinal duplication mesenteric liposarcoma, ovarian tumor, lymphangioma, lipoblastoma, lymphoma and neuroblastoma [9, 6]. In our patient, ultrasonography and CT scan preoperatively suggested that the mass was lipoma and the diagnosis was confirmed by microscopic examination.

Depending on its size, site and the presence of complications, complete excision of the lipoma alone or with the adjacent bowel is the performed treatment [10]. In our case, enucleation of the lipoma was performed without excision or injury to the adjacent intestine, and the appearance of the adjacent bowel and blood supply were normal.

## CONCLUSION

Lipoma is a benign mass that is very rarely found in the mesentery of the jejunum. Histopathology is the definitive diagnosis, and even though the mass is asymptomatic, complete excision is the preferred option.

## CONFLICT OF INTEREST STATEMENT

None declared.

## FUNDING

None.
